# Insecticidal Properties of *Ocimum basilicum* and *Cymbopogon winterianus* against *Acanthoscelides obtectus*, Insect Pest of the Common Bean (*Phaseolus vulgaris*, L.)

**DOI:** 10.3390/insects10050151

**Published:** 2019-05-25

**Authors:** Álvaro Rodríguez-González, Samuel Álvarez-García, Óscar González-López, Franceli Da Silva, Pedro A. Casquero

**Affiliations:** 1 Grupo Universitario de Investigación en Ingeniería y Agricultura Sostenible (GUIIAS), Instituto de Medio Ambiente Recursos Naturales y Biodiversidad. Universidad de León, Avenida de Portugal 41, 24071 León, Spain; salvg@unileon.es (S.Á.-G.); ogonl@unileon.es (Ó.G.-L.); pacasl@unileon.es (P.A.C.); 2 Universidad Federal de Reconcavo de Bahia, Rui Barbosa 710, CEP 44380-000 Cruz das Almas, Brazil; franceli@ufrb.edu.br

**Keywords:** stored bean pest, essential oils, development, emergence, bean damage

## Abstract

The bean weevil, *Acanthoscelides obtectus* Say (Coleoptera: Chrysomelidae: Bruchinae), causes severe post-harvest losses in the common bean, *Phaseolus vulgaris* L. The control of this insect is still poor and involves the use of conventional insecticides. There is an increasing demand in the search for new active substances and products for pest control towards reduction of adverse effects on human health and the environment. The protection of grains with alternative products, such as essential oils, is a possible alternative to meet the needs described above. Therefore, this investigation evaluated the applications of basil, *Ocimum basilicum*, and citronella, *Cymbopogon winterianus*, essential oils for *A. obtectus* control. These essential oils significantly reduced the bean weight losses and the number of beans damaged by *A. obtectus* at higher doses than 60 or 120 μL/sample. The number of holes per bean did not differ between the doses of basil essential oil, not even at the dose of 60 μL, while it was higher at 120 μL, probably due to a lower capacity of movement of the insects treated with this dose and/or the oil’s direct or indirect effects on the insects. Basil and citronella oils exhibited similar patterns of insecticidal activity over the insect, both directly in adult insects or indirectly over bean seeds. These essential oils affected the development of *A. obtectus* since the greatest doses applied on beans decreased the emergence of the bean weevil. The results prove the insecticidal capacity of the tested essential oils and hence their potential as active substances against *A. obtectus* in environmentally low risk pest control strategies. Supplementary trials should be conducted under real storage conditions.

## 1. Introduction

The bean weevil, *Acanthoscelides obtectus* Say (Coleoptera: Chrysomelidae: Bruchinae), is a pest that thrives primarily in stored common beans, *Phaseolus vulgaris* L. (wild and cultivated) [1,2,3]. *A. obtectus* attacks *P. vulgaris* seeds while they are still in the field, and the damage continues during storage, where it causes the greatest losses [4]. According to [5], when not treated, *A. obtectus* population grows exponentially causing the loss of whole crops within a few months.

The control and management of this pest in big storage facilities relies mainly on the use of phosphine, pyrethroids, organophosphates, and other synthetic insecticides [6], but these products are highly toxic to human health and the environment, to which is added the problem of being able to develop resistances on the part of the insects against these products [7]. Control applied by small farmers is virtually non-existent. As a result, research focuses on the development of new compounds with greater selectivity, less environmental persistence, and a variety of modes of action and new sustainable alternatives [8,9].

Previous research has suggested that finding new compounds with greater selectivity, less environmental persistence, and a variety of modes of action is the key for a new generation of control strategies [9]. Plant metabolites and essential oils possess these characteristics and pose substantially fewer risks than those of traditional chemical insecticides. Therefore, natural plant products have been proposed worldwide as an alternative for the control of mites and insect pests [10,11,12,13,14,15], including *A. obtectus* [16,17]. Studies have shown the insecticidal potential of essential oils and their capacity to disrupt insect development [18,19] through interference of the insect nervous system: andaminergic transmissions [20,21,22,23], GABAnergic transmissions [24,25,26], and the inhibition of acetylcholinesterases [27,28,29].

*C. winterianus* extracts have shown repellency and substantial control capacity against many species from different insect orders, such as *Spodoptera exigua* Hübner (Lepidoptera: Noctuidae) [30,31], *Anopheles gambiae* (Insecta: Culicidae) [32], and *Callosobruchus maculatus* Fabricius (Coleoptera: Chrysomelidae: Bruchinae) [33], the former being an important stored product pest. On the other hand, *O. basilicum* has confronted several storage pests, such as *Sithopilus zeamais* Motschulsky (Coleoptera: Curculionidae) [34] and *Rhyzoperta dominica* Fabricius (Coleoptera: Bostrichidae) [35], but does not show insecticidal activity against Coleoptera, a species belonging to other insect orders, such as *S. exigua* [31].

The present study explores the insecticidal potential of *O. basilicum* and *C. winterianus* essential oils against *A. obtectus* on stored beans by non-fumigant applications. Moreover, research on the biological activity of essential oils against the bean weevil during storage is extrapolated to the effects on the behavior of the adult insects and their development.

## 2. Materials and Methods

### 2.1. Insects Rearing

The original population of *A. obtectus* adults was collected during the year 2013 in different bean storage facilities, all of them located in the Protected Geographical Indication (PGI) “Alubia de La Bañeza-León,” which certifies the quality and high standards of beans from this region (EC Reg. n.256/2010 published on 26 March 2010, OJEU L880/17). The insects were reared in glass jars (150 mm in diameter and 250 mm high) with common bean (*Phaseolus vulgaris*) seeds and covered with cloth, allowing gas exchange. Every three days all *A. obtectus* adults were removed from the jar in order to maintain a population of young adults (1 to 3 day old) ready to use in the experiments. *A. obtectus* adults, before and after the treatments, were kept in a chamber with a controlled temperature (24 ± 1 °C), humidity (60 ± 5%), and a photoperiod of 16 h of light (luminous intensity of 1000 lux) and 8 h of darkness.

### 2.2. Essential Oils

*O. basilicum* and *C. winterianus* Java type essential oils obtained by hydrodestillation were purchased from Naissance (Neath, Wales). Volatile components of essential oils were identified by gas chromatography (GC) according to the information provided by the company described above. Analysis for the main compounds are depicted in Table 1.

### 2.3. Design of Experiments

#### 2.3.1. Experiment 1: Essential Oil Effects on *A. obtectus* Adults

This bioassay was conducted to determine the dose-dependent toxicity of *O. basilicum* and *C. winterianus* essential oils against *A. obtectus* adults. For the treatments application, a Potter Tower (Burkard Scientific Limited, Uxbridge, UK) [36] of manual loading coupled to an air compressor was used. The total volume used in each spray was 1 mL, applied on Petri dishes (90 mm in diameter) covered with a sterile filter paper (Sigma-Aldrich Chemie GmbH, Steinheim, Germany), to make sure that the treatments were retained, at 40 kPa. Five doses (6, 12, 24, 60, and 120 μL/petri dish) of *O. basilicum* and *C. winterianus* were diluted in ethanol, and four replicates were performed for each of them. A treatment with ethanol (without oil) was used as a control. After application of treatments, twenty unsexed 1 to 3 day old *A. obtectus* were placed in the Petri dish. From now, ‘Petri dish’ will be considered as the basic test unit. The Petri dishes were kept in a chamber with a controlled temperature (24 ± 1 °C), humidity (60 ± 5%), and a photoperiod of 16 h of light (luminous intensity of 1000 lux) and 8 h of darkness. On the covers of Petri dishes, 8 holes of 1 mm diameter (8 mm^2^) were made to avoid the vapor accumulation effect from the treatments. Daily monitoring was carried out during the following 15 days after the application of each dose of essential oil, counting the mortality of *A. obtectus* adults.

#### 2.3.2. Experiment 2: Essential Oil Effects on the Development of *A. obtectus*

The second bioassay was conducted to determine the toxicity (sublethal effects) of the essential oils against *A. obtectus* adults when sprayed over *P. vulgaris* seeds. The same doses of each essential oil as described above were used. The treatments were applied with a Potter Tower (Burkard Scientific Limited, Uxbridge, UK) [36] over 70 g of bean seeds that were placed in 0.33 L glass jars (80 mm in diameter and 85 mm in height). After the application, the jars were manually shaken for 60 s, ensuring a complete distribution of the essential oil over the beans. After the treatment’s application, twenty unsexed 1 to 3 day old *A. obtectus* adults were placed in each jar, and the jars were kept under controlled conditions of temperature (24 ± 1 °C), humidity (60 ± 5%), and a photoperiod of 16 h of light (luminous intensity of 1000 lux) and 8 h of darkness. On the covers of the glass jars, 8 holes of 1 mm diameter (8 mm^2^) were made to avoid the vapor accumulation effect from the treatments. Daily monitoring was carried out during the following 15 days after the application of each treatment, counting the mortality of *A. obtectus* adults. Five doses (18, 36, 72, 180 and 360 μL/litre) of *O. basilicum* and *C. winterianus* were diluted in ethanol. A treatment with ethanol was used as a control. Four replicates were used for each dose, and the doses were calculated as μL of essential oil/volume of the container. From day 32 after the treatment application on the beans, the number of *A. obtectus* that emerged from the beans (first generation) was recorded. Likewise, from day 48, the number of damaged beans (with at least one hole), holes per bean, and the weight loss of the damaged beans were also recorded in this experiment.

### 2.4. Statistical Analysis

**Figure 1:**
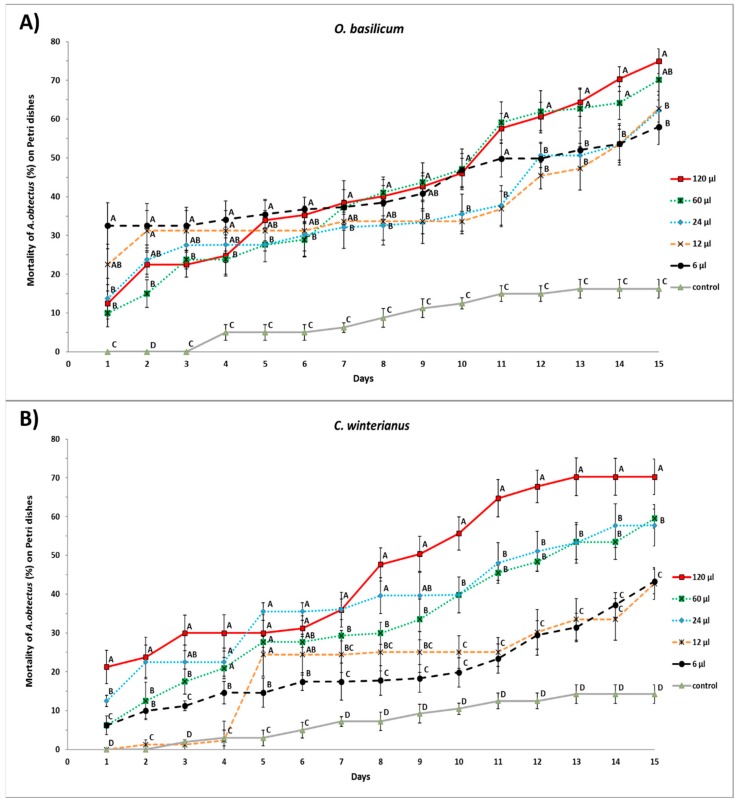
Accumulated mortality of *A. obtectus* on Petri dishes exposed to different doses of *O. basilicum* (**A**) and *C. winterianus* (**B**) essential oils. Different capital letters indicate significant differences among doses and control for the same day; least significant difference (LSD) test at 0.05. The symbols represent the mean of four replicates for each dose. Vertical bars represent the Standard Error (SE) of the mean.

Experiment 1. A randomly completed experiment Generalized Linear Model (GLM) procedure, with five doses for each essential oil and four replicates, was subjected to ANOVA (data means were normally distributed and presented homocedasticity). Differences (*p* < 0.05) on the same day among doses (within the same essential oil), and the control, were examined by mean comparisons using the Least Significant Difference (LSD) test. The mortality data were corrected with the Abbott’s formula [37] in the experiment described. Mean values and standard errors are given in Figure 1 (Mortality of *A. obtectus* on Petri dishes).

**Figure 2:**
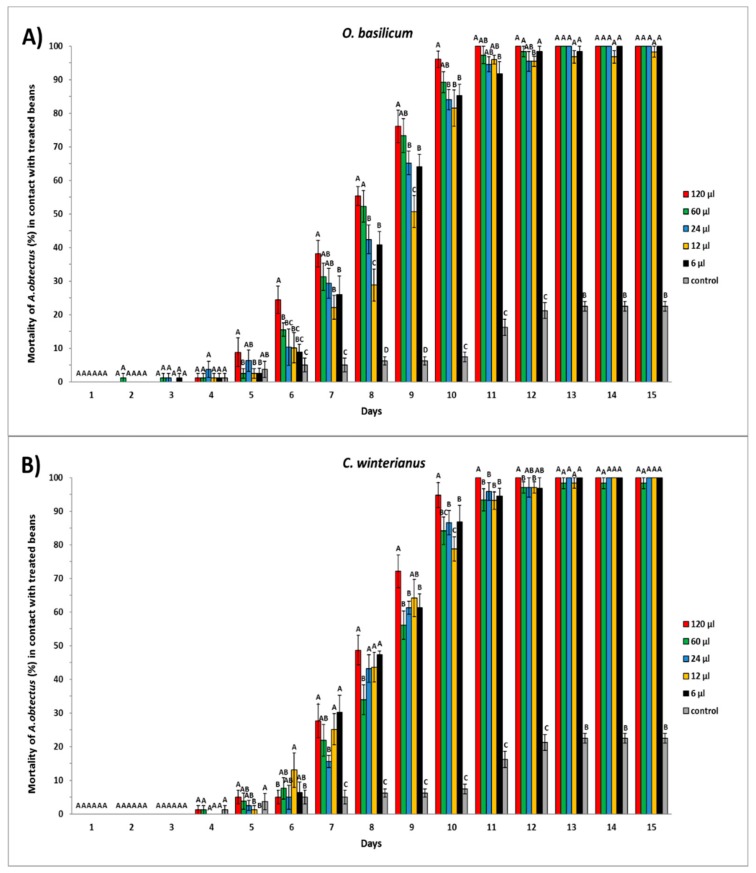
Mortality of *A. obtectus* in 0.33 L/glass jars exposed to beans treated with different doses of *O. basilicum* (**A**) and *C. winterianus* (**B**) essential oils. Different capital letters indicate significant differences among doses and control for the same day; LSD test at 0.05. The symbols represent the mean of four replicates for each dose. Vertical bars represent the Standard Error (SE) of the mean.

Experiment 2. A randomly completed experiment Generalized Linear Model (GLM) procedure, with five doses for each essential oil and four replicates, was subjected to ANOVA (data means were normally distributed and presented homocedasticity). Differences (*p* < 0.05) among insecticidal activity on beans, accumulated emergence, bean weight loss, number of damaged beans, and number of holes by beans among the doses (within the same essential oils), and the control, were examined by mean comparisons using the Least Significant Difference (LSD) test. Mean values and standard errors are given in Figure 2 (insecticidal activity on beans), Figure 4 (bean weight loss) and Table 2 (number of damaged beans and number of holes by bean).

## 3. Results

### 3.1. Mortality of A. obtectus Adults against Different Doses of Essential Oils (Experiment 1)

Figure 1A,B show significant differences among doses of essentials oils when they were applied directly on *A. obtectus* adults placed in Petri dishes.

For *O. basilicum* essential oil, the best control was achieved for the dose 120 μL on day 15 after application, killing 74.94 ± 3.19% of the adults evaluated. This value was significantly greater (F = 25.31; df = 5.18; *p* < 0.0001) than the obtained for the other doses tested, except when applying 60 μL of essential oil (70.08 ± 5.17% of mortality). The doses of 24 and 12 μL were able to kill around 62% of adults on day 15th, not significantly greater than the mortality obtained by the 6 μL dose, which was 58.02 ± 4.58% on day 15th. The control treatment significantly differed from the 1st day after application, in which percentage of mortality was 16.25 ± 2.93% on day 15th (Figure 1A).

Likewise, the best control capacity obtained for *C. winterianus* essential oil was achieved for the dose 120 μL on day 15 after application, killing 70.25 ± 4.58% of the adults evaluated. This value was significantly greater (F = 15.73; df = 5.18; *p* < 0.0001) than for the other doses. The doses 60 and 24 μL were able to kill 59.50 ± 2.46 and 57.75 ± 5.39% of adults respectively, significantly higher than the mortality obtained for the 12 μL and 6 μL doses (43.28 ± 3.19 and 42.68 ± 4.10% of adults died with these doses on day 15th). Control treatment differed significantly from the 5th day after application, in which percentage of mortality was 14.25 ± 2.39% on day 15th (Figure 1B).

### 3.2. Effect of Essential Oils on the Development of A. obtectus (Experiment 2)

#### 3.2.1. Persistence of Insecticidal Activity on Beans

Significant differences were observed during the first days of the evaluation over the insect mortality when beans were treated with different doses of *O. basilicum* and *C. winterianus* essential oils.

On the 6th day of the evaluation for the test carried out using *O. basilicum*, the 120 μL dose was able to kill 24.43 ± 4.06% of the initial insect population, significantly greater (F = 2.78; df = 5.18; *p* = 0.049) than the mortality obtained by rest of doses evaluated. Meanwhile, on the 8th day, the 60 μL and 120 μL doses were able to kill more than 50% of the adult insects, being significantly higher (F = 9.04; df = 5.18; *p* < 0.0001) than the control achieved by 24, 12, and 6 μL doses.

Finally, the 120 μL dose caused a total control (100% of insects died) from the 11th day of evaluation, whereas beans treated with 60 and 24 μL doses achieved the total control of the insects only from day 13th. From the 7th day onwards, mortality achieved by all doses was significantly higher than the control treatment, in which percentage mortality was 22.50 ± 1.44% on day 15th (Figure 2A).

As regards *C. winterianus* essential oil, on the 8th day of evaluation, the 120 μL dose was able to kill 48.66 ± 4.41% of the evaluated insect population, only significantly greater (F = 7.48; df = 5.18; *p* < 0.0001) than the obtained by 60 μL dose. Nevertheless, on day 9, a 120 μL dose was able to kill more than 72% of the insects, being significantly greater (F = 20.09; df = 5.18; *p* < 0.0001) than the obtained by other doses evaluated, with the exception of 12 μL (64.18 ± 5.59%). Finally, when using a 120 μL dose, a total control (100% of insects died) was achieved from day 11th. Doses of 24 and 6 μL, were able to kill 100% of the insects only from day 13. Again, mortality observed for all doses was significantly higher than the control treatment from the 7th day onwards, in which the percentage mortality was 21.60 ± 1.93% on day 15th (Figure 2B).

#### 3.2.2. Accumulated Emergence of *A. obtectus* Adults from Beans Treated with Different Doses of Essential Oils

Different doses of *O. basilicum* and *C. winterianus* essential oils applied over beans affected the bean weevil emergence. The emergence period began 32 days after the application of the oils, and concluded 16 days later, on day 48.

**Figure 3:**
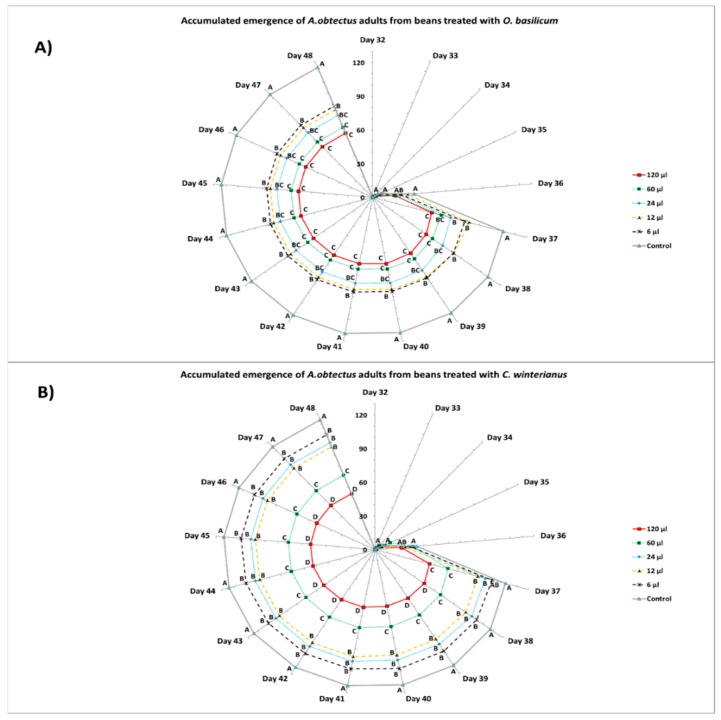
Accumulated emergence (mean ± SE) of *A. obtectus* adults in 0.33 L/glass jar from beans treated with different doses of *O. basilicum* (**A**) and *C. winterianus* (**B**) essential oils. Different capital letters indicate significant differences among doses and control for the same day; LSD test at 0.05.

The total emergence of bean weevil adults from beans treated with any of *O. basilicum* doses on the 37th day of evaluation was significantly lower (F = 2.98; df = 5.18; *p* = 0.039) than in the control treatment (110.25 ± 5.14). In the last day of emergence (day 48), the treatment of beans with 120 μL and 60 μL reduced significantly (F = 3.00; df = 5.18; *p* = 0.045) the number of insects that emerged (61.25 ± 4.31 and 66.75 ± 4.96, respectively) compared to the treatment 12 μL, 6 μL doses and the control (Figure 3A).

The insect emergence from beans treated with any of the *C. winterianus* doses applied was significantly lower (F = 7.12; df = 5.18; *p* = 0.018) than the results obtained in the control treatment (110.25 ± 4.55) on the 37th day of evaluation. In the last day of emergence (day 48), the treatment of beans with 120 μL dose significantly reduced (F = 4.27; df = 5.18; *p* = 0.026) the number of insects that emerged (53.50 ± 3.40) compared to the rest of doses (60, 24, 12, 6 μL) and the control (Figure 3B).

#### 3.2.3. Bean Damage

Applications of different doses of *O. basilicum* and *C. winterianus* over beans repelled *A. obtectus* attack, significantly reducing the number of beans affected (beans damaged) by the insects after treatment with *O. basilicum* (F = 13.29; df = 5.18; *p* < 0.0001) and *C. winterianus* (F = 5.93; df = 5.18; *p* = 0.002) essential oils (Table 1).

The number of holes per bean was significantly higher for the beans treated with 120 μL of the basil essential oil (F = 2.60; df = 5.18; *p* = 0.048) compared to those treated with the lower doses (except the 60 μL dose) and the control. There were no significant differences in the number of holes per bean after the application of the different doses of *C. winterianus* and the control treatment (Table 2).

**Table 2:** Number of damaged beans (mean ± SE) and number of holes per bean (mean ± SE) caused by *A. obtectus* adults that emerged from 70 g of beans treated in 0.33 L/glass jars with different doses of *O. basilicum* and *C. winterianus* essential oils.

Essential Oil	Dose (µL)	Number of Beans Damaged (with at Least One Hole)	Number of Holes Per Bean
*O. basilicum*	Control	51.50 ± 4.99 a ^1^	2.09 ± 0.67 b ^1^
	6	36.75 ± 3.81 b	2.40 ± 0.18 b
	12	36.00 ± 3.39 b	2.36 ± 0.35 b
	24	25.00 ± 4.63 c	2.82 ± 0.63 b
	60	23.25 ± 2.13 cd	2.98 ± 0.46 ab
	120	13.50 ± 1.55 d	4.90 ± 1.21 a
	F	13.299	2.304
	df	5.18	5.18
	P	≤0.000	0.088
*C. winterianus*	Control	51.50 ± 4.99 a ^1^	2.09 ± 0.67 a ^1^
	6	51.00 ± 3.39 a	2.15 ± 0.08 a
	12	45.25 ± 3.92 ab	2.33 ± 0.11 a
	24	41.00 ± 3.85 ab	2.23 ± 0.35 a
	60	36.50 ± 3.66 bc	1.92 ± 0.27 a
	120	23.75 ± 5.48 c	2.09 ± 0.26 a
	F	5.935	0.154
	df	5.18	5.18
	P	0.002	0.976

^1^ Different lowercase letters indicate significant differences among beans treated by different doses of essential oils and control; LSD test at 0.05.

#### 3.2.4. Bean Weight Losses

**Figure 4:**
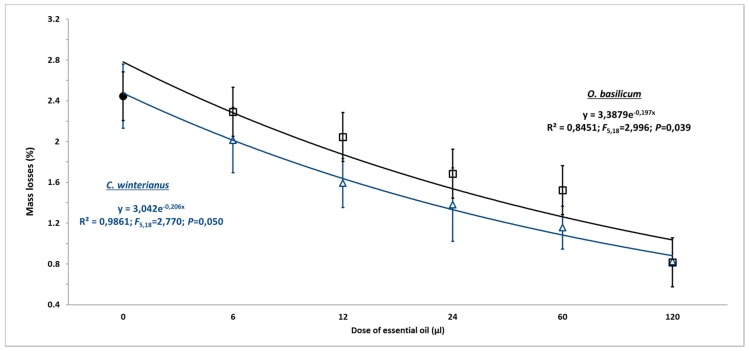
Bean weight losses caused by *A. obtectus* in 0.33 L/glass jar on the absence (control) (●) and the presence of increasing doses of *O. basilicum* (**□**) and *C. winterianus* (**Δ**) essential oils. The symbols represent the mean of four replicates for each dose of essential oils. Vertical bars represent the Standard Error of the mean (SE).

Beans incubated with *A. obtectus*, which were previously treated with different doses of *O. basilicum*, showed weight losses ranging between 0.81 ± 0.21% (120 μL dose) and 2.29 ± 0.31% (6 μL dose), while beans under a control treatment (2.44 ± 0.64%) had a weight loss significantly higher (F = 2.99; df = 5.18; *p* = 0.039) than the rest of the doses evaluated (Figure 4).

Finally, beans incubated with *A. obtectus*, which were previously treated with different doses of *C. winterianus*, showed weight losses ranging between 0.83 ± 0.31% (120 μL dose) and 2.01 ± 0.38% (6 μL dose), while beans under the control treatment (2.44 ± 0.64%) had weight loss significantly higher (F = 2.77; df = 5.18; *p* = 0.050) than the rest of doses evaluated (Figure 4).

## 4. Discussion

The essential oils do not constitute a threat to the environment or to human health, and so they have been portrayed as a possible alternative to synthetic insecticides [38]. The essential oils are volatile hydrocarbon mixtures, which have a variety of functional groups [31]. These oils are derived from secondary plant metabolism and play a key role in their development, both directly, through the defense of the plant against microorganisms and herbivores [31]. Many authors [39,40,41] have described the essential oils to be composed of substances from various chemical groups, such as terpenes (monoterpenes and sesquiterpenes), phenylpropanoids, alcohols, esters, aldehydes, ketones, among others. The biological activity of essential oils depends not only on their chemical composition, but also on the concentration and proportion of these substances [39,40,41].

The essential oil of *O. basilicum* was principally composed by the aromatic ether Estragol (74.0%) and the terpene with alcohol group Linalool (17.8%), which are known to have repellent and toxic activities against stored product insect [42]. The proportion of main components obtained in *O. basilicum* oil, were similar to those described by Kim et al. [43] and Souza et al. [35]. The genus *Ocimum*, belonging to the family Lamiaceae, has been investigated with regard to its insecticidal properties against diverse insect pests [35,44,45,46].

The main components for *C. winterianus* were the terpenoid mix Citral (0.7%), the monoterpenoids Citronellol (11.5%) and Citronellal (34.0%), the monoterpenoid with an alcohol Geraniol (22.0%), and the terpene Limonene (3.5%), coinciding with those described by Leite et al. [47] and Rodrigues et al. [48]. That is, the main essential oil components of *C. winterianus* oil are the monoterpenes, citronellol, citronellal and geraniol. Monoterpenes possess insecticidal and insect repellent properties, as described by Isman [49], Silva et al. [30] and Chen and Viljoen [50]. The proportion of main components obtained in *C. winterianus* oil were very similar to those obtained by Renkema et al. [51], Martins et al. [52], and Vieira and Simon [53] described that the chemical composition of essential oils can show large variability, both interspecific and within the same species. It seems to depend on the genetic characteristics of the plant and on the conditions under which it was grown.

These essential oils exhibited similar patterns for insecticidal activity over the *A. obtectus* when sprayed directly in Petri dishes or indirectly over beans in glass jars using different doses. The insecticidal activity of both oils did not exceed 15 days after application. In the same range, it is well described by Ilboudo et al. [54] for several other essential oils. Losses of activity for essential oils are normally due to degradation of the active compounds. In this respect, essential oils containing more hydrogenated compounds are more susceptible to oxidation [55]. Various studies with essential oils obtained from species of the genus *Ocimum* spp. (*O. basilicum* and *O. gratissimum*) showed good results regarding their insecticidal effect against insect pests that attack grains. Kéita et al. [44] evaluated (by fumigation) the effect of *O. basilicum* and *O. gratissimum* for the control of *Callosobruchus maculatus* F. (Coleoptera: Chrysomelidae: Bruchinae) and obtained 80% and 70% mortality of the insect population evaluated with 25 μL. Rozman et al. [42] reported toxicity of fumigated linalool against *Tribolium castaneum* Herbst (Coleoptera: Tenebrionidae), *Rhyzopertha dominica* (Coleoptera: Bostrichidae), and *Sitophilus oryzae* (Coleoptera: Curculionidae), in fumigation with Linalool, one of the main components of basil essential oil, where Linalool was highly effective for *R. dominica*, and caused 100% mortality at the lowest tested concentration (0.1 mL/720 mL of volume). Ogendo et al. [46] obtained a 98%, 99%, and 100% mortality against *R. dominica*, *Oryzaephilus surinamensis* L. (Coleoptera: Silvanidae) and *Callosobruchus chinensis* L. (Coleoptera: Chrysomelidae) using 1 μL of *O. gratissimum* essential oil per litre of air.

Other studies have shown diverse activities and effects of *C. winterianus*, such as its use an insect repellant [56], its larvicidal effect for certain mosquito species [57], and its insecticidal against *Frankliniella schultzei* Trybom (Thysanoptera: Thripidae) and *Myzus persicae* Sulzer (Homoptera: Aphididae) species [58]. Citronella oil caused also repellency on *C. maculatus* (Fabr) adults [33] and feed deterrence and larval mortality on *S. frugiperda* Walker (Lepidoptera: Noctuidae) [59].

*A. obtectus* accumulated emergence decreased with an increase in the doses of both essential oils. This phenomenon can be due to a lower capacity of movement of the insects treated with this dose and/or the oil’s direct or indirect effects on the insects (such as repellency and/or anti feeding activity) at one or more of the insect life stages (egg, larva, pupa, and adult), as have been described by Papachristos and Stamopoulos [60] and Schmidt and Streloke [61].

Several authors [62,63,64] have described different activities of essential oils on eggs and some insect adults. Specifically, for this insect pest, *A. obtectus*, developmental traits, such as adult life span, the ability of larvae to enter the bean, and larva/pupa susceptibilities have been shown to be differentially affected by different essential oils [18,65].

Basil and citronella oils exhibited significant reductions on bean weight and the number of beans damaged by *A. obtectus* when greater doses were applied over beans. In addition, the high doses (60 and 120 μL) of basil oil generated a greater concentration of holes in the damaged beans, showing the toxic activity of this essential oil through its Linallol component [42]. The behavior and/or repellency shown by *A. obtectus* towards treated beans has also been observed by Rodríguez-González et al. [66] using biological control agents.

## 5. Conclusions

*O. basilicum* and *C. winterianus* essential oils significantly reduced the bean weight losses caused by *A. obtectus*, and significantly reduced the number of beans damaged when doses of 60 or 120 μL were applied to them, in addition to concentrating a greater number of holes per bean with the 120 μL dose of *O. basilicum.* In addition, these essential oils exhibited similar patterns for insecticidal activities over the insects when these oils were sprayed directly in Petri dishes or indirectly over beans in glass jars. These essential oils affected the development of *A. obtectus*, since the greatest doses applied on beans decreased the emergence of the bean weevil. The present study supports the insecticidal capacity of basil and citronella essential oils against *A. obtectus* and their potential use in environmentally low risk pest control strategies. Supplementary trials should be conducted under real storage conditions

## Reference

## Figures and Tables

**Table 1:** Composition of basil and citronella essentials oils obtained with gas chromatographic analysis.

Essential Oil	Compounds (%)	Total (%)
*O. basilicum*	Citral (0.70)	71.70
	Citronellol (11.50)	
	Citronellal (34.00)	
	Geraniol (22.00)	
	Limonene (3.50)	
*C. winterianus*	Estragol (74.00)	91.80
	Linalool (17.80)	
